# Identification of watermelon heat shock protein members and tissue-specific gene expression analysis under combined drought and heat stresses

**DOI:** 10.3906/biy-1907-5

**Published:** 2019-12-13

**Authors:** Yasemin Çelik ALTUNOĞLU, Merve KELEŞ, Tevfik Hasan CAN, Mehmet Cengiz BALOĞLU

**Affiliations:** 1 Department of Genetics and Bioengineering, Faculty of Engineering and Architecture, Kastamonu University, Kastamonu Turkey

**Keywords:** *Citrillus lanatus*(watermelon), heat shock protein, abiotic stress, bioinformatics analysis, gene expression analysis

## Abstract

Heat shock protein (*Hsp) *gene family members in the watermelon genome were identified and characterized by bioinformatics analysis. In addition, expression profiles of genes under combined drought and heat stress conditions were experimentally analyzed. In the watermelon genome, 39 genes belonging to the sHsp family, 101 genes belonging to the Hsp40 family, 23 genes belonging to the Hsp60 family, 12 genes belonging to the Hsp70 family, 6 genes belonging to the Hsp90 family, and 102 genes belonging to the Hsp100 family were found. It was also observed that the proteins in the same cluster in the phylogenetic trees had similar motif patterns. When the estimated 3-dimensional structures of the Hsp proteins were examined, it was determined that the α-helical structure was dominant in almost all families. The most orthologous relationship appeared to be between watermelon, soybean, and poplar in the *ClaHsp* gene families. For tissue-specific gene expression analysis under combined stress conditions, expression analysis of one representative *Hsp* gene each from root, stem, leaf, and shoot tissues was performed by real-time PCR. A significant increase was detected usually at 30 min in almost all tissues. This study provides extensive information for watermelon Hsps, and can enhance our knowledge about the relationships between *Hsp *genes and combined stresses.

## 1. Introduction

*Citrillus lanatus* (watermelon) is an important plant in the family Cucurbitaceae, constituting 7% of the world’s total fields devoted to crop production. The world annual watermelon (*Citrillus lanatus*) production is about 90 million tons, and watermelon (*Citrillus lanatus*) is among the 5 most consumed fresh fruits (FAO, u1d42). Although watermelon (*Citrillus lanatus*) is mostly composed of water (up to 90%), it contains important nutritious compounds such as sugar, lycopene, and health-promoting amino acids including citrulline, arginine, and glutathione (Hayashi et al., 2005; Perkins-Veazie et al., 2006; Collins et al., 2007; Guo et al., 2013). 

Heat-shock proteins (Hsps) are a special protein family that are synthesized in response to a variety of stress conditions including high temperatures, and are required for the growth and survival of the cell (Whitley et al., 1999; Kumar et al., 2012; Çelik Altunoğlu, 2016). Hsps are functional in many processes such as protein folding, cellular regulation, and inhibition of the accumulation of inappropriate proteins in the cell. In addition, members of this protein family are known to be synthesized under different stress conditions and behave like molecular chaperones, which enable proteins to become three-dimensional structure by folding (Henle et al., 1998). They are also key determinants of quality control and have an important role in protecting overall cellular protein balance (Kumar et al., 2012).

Hsps with molecular weights ranging from 10 kDa to 200 kDa are classified according to their molecular weights and functioning mechanisms. They can be divided into 6 classes: sHsp (small heat shock proteins), Hsp40 (J-proteins), Hsp60 (chaperonins), Hsp70, Hsp90, and Hsp100 (Clp proteins). sHSPs that are not present in plant cells under physiological conditions are stimulated by stresses including drought, salinity, oxida species, and low temperatures (Sun et al., 2002). Hsp40 is characterized by a standard J-domain. J-protein heat shock family members are found everywhere in prokaryotes and eukaryotes at various subcellular sites. J-proteins stand for a large family of molecular chaperones that serve to determine the cellular functions of Hsp70s (Rajan and D’Silva, 2009). The Hsp60 family, found in plastids, mitochondria, and cytoplasm of all eukaryotes and eubacteria, are molecular chaperones that are among the most conserved and found everywhere (Martin et al., 1992). Hsp70 is involved in various cellular functions such as protein transport between membranes, modulation of protein activity, protein folding, regulation of protein degradation, and inhibition of irreversible protein aggregation (Su and Li, 2008). Hsp90 is one of the most abundant proteins in eukaryotes. Unlike other chaperones, Hsp90 is not effective for new protein folding. At a later stage of folding, it binds to substrate proteins that are close to natural (Young et al., 2001). Hsp100/Clp is a class of molecular chaperones that have the ability to resolve almost every protein which has collected after severe stress. They are not necessary under normal growth conditions, and are triggered by extreme heat or other severe stresses (Lund, 2001).

Under both natural and agricultural conditions, plants are often exposed to environmental stresses (Büyük et al., 2012). Abiotic stresses including drought, salinity, extreme temperatures, chemical toxicity, and oxidative stress create serious threats to agriculture and cause deterioration of the natural environment (Wang et al., 2004). Drought is one of the most significant abiotic stress factors that decreases crop yield with soil yield (Baloğlu et al., 2014a). Drought causes the stoma closure associated with reduced CO2 intake in leaves; this results in instabilities between photosynthetic electron transports and carbon assimilation (Kosova et al., 2018). High temperature influences the metabolism and structure of plants, especially cell membranes of plants, and many basic physiological processes including photosynthesis, respiration, and water relations (Al-Whaibi, 2011). The combination of drought and heat stress has been found to have more detrimental effects on the growth and productivity of plants compared to each of the different stresses applied separately. For example, when plants encounter heat stress, they open their stomata to cool their leaves by transpiration. When heat stress is combined with drought, the temperature of the leaves is higher because the plants cannot open their stomata. Similar problems will occur when salinity or heavy metal stress is combined with heat stress, because more transpiration leads to more salt or heavy metal intake. Since most of the abiotic stress studies performed in laboratory conditions do not fully reflect the conditions of the natural environment, there may be differences between the results of these studies and the conditions required for the development of highly tolerant plants in natural conditions. This difference explains why transgenic plants with high tolerance to abiotic and biotic stress under laboratory conditions may not show high tolerance in the natural environment. Molecular, physiological, and metabolic examination of the combination of stressors facilitates the elimination of this difference and the development of plants that are more tolerant to natural stress conditions (Mittler, 2006). Since plants in their natural environment are often exposed to combined stresses such as drought and high temperature, researching the response of plants to combinations of stress factors can help resolve stress tolerance mechanisms in plants.

Complete genomic data of organisms are useful for determining important gene families using bioinformatics methods. Hsps have been characterized in many plant species, including *Arabidopsis *(Swindell et al., 2007), wheat (Muthusamy et al., 2017), sunflower (Büyük et al., 2012), rice (Singh et al., 2010; Jiang et al., 2014; Wang et al., 2014), tomato (Zai et al., 2017), poplar (Yer et al., 2016; Yer et al., 2018), and eucalyptus (Altunoğlu, 2016). The complete genome sequence of watermelon was published by Guo et al. in 2013; however, to our knowledge, Hsp family members have not yet been defined in the watermelon genome. In the current study, watermelon *Hsp* genes have been identified and characterized. In addition, expression profiles of genes under combined drought and heat stress conditions were experimentally analyzed and the results were compared using bioinformatics.

## 2. Materials and methods

### 2.1. Identification of heat shock protein genes (hsp) in watermelon genome

According to our previously published study (Baloğlu, 2014b; Baloğlu, 2014c; Kavas et al., 2015; Kavas et al., 2016), different search strategies were performed to elucidate *Hsp* genes from the watermelon genome. First, using the HSPIR (Heat Shock Protein Information Resource) database, the genomic, coding region, and protein sequences of *Hsp* genes were obtained for all plants. For these sequences, a BLASTP (Protein Blast Sequence Comparison) search was performed using the Cucurbit Genomics database. In addition, defined sequences were scanned and selected according to the protected regions by using the Hidden Markov Model (HMM). Protected regions of the selected sequences were checked with the Pfam (https://pfam.xfam.org/) database, and they were retrieved for the study as watermelon Hsps.****Instability index, molecular weights, and isoelectronic effect values (pI) of these Hsp sequences were obtained using the ProtParam tool (u1d45).

### 2.2. Determination of chromosomal locations and estimation of gene structure of hsps

Chromosomal localizations of the *Hsp* genes found in watermelon plants were made using the Cucurbit Genomics database (u1d46); these localizations were then shown on chromosomes with the MapChart program. To clarify the structure of the watermelon *Hsp* genes, the exon–intron regions of the genes were determined by using the Gene Structure Display Server (http://gsds.cbi.pku.edu.cn/) (Hu et al., 2015).

### 2.3. Sequence alignment, phylogenetic analysis, and identification of preserved motifs

Multiple sequence alignment of the defined watermelon Hsps was performed using ClustalW via the MEGA7 program. Phylogenetic trees were drawn using sequences aligned with the ClustalW program. Maximum likelihood method (Milligan, 2003) was used when the phylogenetic tree was drawn. Through this method, a phylogenetic tree was formed with 1000 repetitive bootstrap analyses. The drawn phylogenetic tree was visualized using the iTOL database, and each cluster was indicated in a different color. The MEME Suit web portal (Bailey et al., 2015) was used to identify the preserved motifs. When defined, the number of motifs was 20 and the width of the motif was chosen as optimum ≥2 and ≤300. The obtained motifs were also scanned using the InterPro database with InterProScan (Quevillon et al., 2005).

### 2.4. Gene ontology analyzes

The Blast2Go program was used for functional analysis of determined watermelon Hsps (Conesa and Götz, 2008). The amino acid sequences of the watermelon Hsps were loaded, and biological and molecular functions and cellular contents were obtained by GO classification by following steps such as BLAST, interpro, mapping, annotation, charting, and graphing.

### 2.5. Computation of homologous and nonhomologous change rates

Multiple amino-acid sequence alignment was performed through the ClustalW program by aligning orthologous gene pairs among the duplicated *Hsp* genes in the watermelon genome and *Arabidopsis thaliana*,* Oryza sativa*,* Glycine max*,* Populus trichocarpa*,* Vitis vinifera*,**and* Zea mays*. Afterwards, homologous (Ks) and nonhomologous (Ka) exchange rates were calculated using the PAL2NAL program by a method which aligns the amino acid sequences of the *Hsp* genes with their original complementary DNA sequences (Suyama et al., 2006). Thus, for each *Hsp* gene, duplications and separation time (million years ago, MYA) were calculated using homologous mutation ratios of λ changes corresponding to each homologous region and year (T = Ks/2λ (λ = 6.5 × 10−9) (Lynch and Conery, 2000; Yang et al., 2008).

### 2.6. Homology modeling of Hsps

A Protein Data Bank (PDB) scan was performed to identify the sequences which were similar to watermelon Hsp proteins and the most suitable sample with a known 3-dimensional structure by using BLASTP (Berman et al., 2000). Possible 3-dimensional structures of the watermelon Hsps were analyzed by using the Phyre2 program with the obtained data (Kelley et al., 2015).

### 2.7. Identification of miRNAs targeted to hsp genes in watermelon

The plant miRNA database was used to identify miRNAs targeting previously known plant miRNA genes using the miRBase v20.0 (http://www.mirbase.org/) program for the identification of miRNA-controlled gene targets (Budak and Akpinar, 2015). All assumed plant and watermelon miRNAs were identified by aligning all known plant miRNAs with *Hsp* gene transcripts in the watermelon plant using the psRNA Target server (http://plantgrn.noble.org/psRNATarget/home) (Dai and Zhao, 2011).

### 2.8. Determination of expression profiles of watermelon hsp genes using transcriptomic data

For RNA-Seq analysis, all Illimuna HiSeq readings were provided through an open database archive called SRA (Sequence Read Archive). Accession numbers: SRR1724899, SRR1724900, SRR1724901, SRR1724902, SRR1724903, SRR1724943 (10th, 18th, 26th, 34th, 42nd, and 50th day fruit flesh collected after pollination, respectively), WM-UR-1/SRR1001435, WM-UR-2/SRR1001436 (white fruit 10 days after pollination), WM-IM-1/SRR1001437, WM-IM-2/SRR1001438 (white–pink fruit 18 days after pollination), WM-PM-1/SRR1001439, WM-PM-2/SRR1001440 (pink fruit 28 days after pollination), WM-MA-1/SRR1001441, WM-MA-2/SRR1001442 (ripe red fruit 34 days after pollination), SRR494474, SRR518988, SRR518988 (phloem tissue), SRR494479, SRR518992, SRR518993 (vascular tissues). The “.sra” raw sequence data of all readings was downloaded and converted to the “fastq” format using the NCBI SRA Toolkit’s fastq-dump command. FastQC analysis was carried out in order to perform quality control on all of the remaining readings to remove the low-quality ones from the obtained readings. Normalization and transformation of all readings were performed with the CLC Genomic Workbench version 11.1 program. A hierarchical clustering map (HeatMap) was drawn using gene expression measurements with the Permut Matrix program.

### 2.9. Growing of watermelon plants and stress applications

Watermelon seeds (Crisby F1) were used in the experiments. These seeds, which were supplied by Monsanto Food and Agriculture Trade Limited Company (Antalya), were washed and kept in distilled water for 2 h. At the end of the 2 h, the seeds were planted in plastic pots containing vermiculite. Diameter and volume of plastic pots used in the study were 93 mm and 400 cc, respectively. After the planting was completed, the pots were irrigated with Hoagland solution (Hoagland and Arnon, 1950) and transferred to the climate cabinet. They were grown at 24 ± 2 °C with a 16 h light and 8 h dark photoperiod in the climate cabinet for approximately 35 days at a light intensity of 400 μmol m–2 s–1, and with relative humidity maintained at 55%–80% by watering with Hoagland solution regularly every day. Drought and temperature stresses were applied to the plants together to see their combined effect. A 20% Hoagland solution containing polyethylene glycol 6000 (PEG-6000) was used for drought stress. For temperature stress, the temperature of the climate cabinet was set at 50 °C. The samples were collected at 0, 30 min, 1 h, and 2 h of stress application as in the study conducted by Baloğlu et al. (2014c; 2014d). Samples collected at the zero hour were used as controls. Four different tissue types were collected from the plants. They were root, stem, leaf, and tendril. All leaves except the first-grown cotyledon leaves were collected for analysis. All of the roots and tendrils that could be obtained were collected. All collected samples were frozen in liquid nitrogen and stored at –80 oC until RNA isolation.

### 2.10. RNA isolation and real time PCR analysis

Total RNA was extracted using a Trizol reagent (Life Technologies Corporation, Thermo Fisher Scientific, Waltham, MA, USA) procedure which has been previously described in our studies (Baloglu et al., 2014d; Çelik Altunoğlu et al., 2016b; Çelik Altunoğlu et al., 2017; Çelik Altunoğlu et al., 2018). Absorbance values were then measured with a spectrophotometer (MultiScanGo, Thermo Fisher Scientific, Waltham, MA, USA) at 260/280 nm wavelengths in order to control the purity and quality of RNA dissolved in DEPC water. DNase (RNase-free, Thermo Fisher Scientific, Waltham, MA, USA) was applied for the purification of the isolated RNA samples completely from DNA residues. An u1d47, Hercules, CA, USA) was used for cDNA synthesis. Primers for *Hsp* genes were determined based on their expression patterns in transcriptome data and were designed using the NCBI Primer Blast tool (u1d48) (Supplementary material 1). Real-time polymerase chain reaction (qRT-PCR) was achieved with a Qiagen Rotor Gene 6000 “Real-Time” PCR device. For reaction, temperature optimized primers and SYBR Green Supermix (Bio-Rad, Hercules, CA, USA) were used. From the cDNA samples, 6 trials (3 biologicals and 3 technical replicates) were used. The components for qRT-PCR reaction were 20 μL in total, including 1 μL forward primer, 1 μL reverse primer (25 pmol/μL), 2 μL cDNA (100 ng/μL), 10 μL SYBR Green Supermix, and water. qRT-PCR conditions were as follows: after first denaturation at 95 °C for 5 min, 1 min at 95 °C, 1 min at 55–58 °C, and 1 min at 72 °C were repeated 35 times. The amplified cDNAs were analyzed by qRT-PCR at 530 nm. In addition, melting curve analysis was performed to verify the correct replication. In qRT-PCR analysis, a reference gene that is known to be unchanged or least affected in expression under various conditions was used for normalization. *Tua* gene (Tubulin alpha chain) was chosen as a reference gene in the study (Kong et al., 2014). Calculated ΔCT (ΔCT = CT sample – CT reference) and ΔΔCT (ΔΔCT = ΔCT stressed sample – ΔCT control) values were used to reveal the differences in gene expression levels, as 2ΔΔCT and graphs of expression profiles were drawn (Livak and Schmittgen, 2001).

### 2.11. Statistical analysis

Statistical calculations and comparisons were made by using the Minitab 18 package program. The difference between stressed samples and control samples is shown as follows: *, P = <0.1; **, P**= <0.05; ***, P = <0.01. Using the univariate-ANOVA test for statistical analysis of the results, it was accepted that the difference was significant when P value was <0.01.

## 3. Results

### 3.1. Determination and characterization of watermelon hsp genes

Hsp sequences from various plant species were obtained from the HSPIR database, and those belonging to watermelon plants were identified using the Cucurbit Genomics database. As a result, 39 genes belonging to sHsp were found and named, from *ClasHsp-01* to *ClasHsp-39*. One hundred and one genes for Hsp40 (*ClaHsp40*), 23 genes for Hsp60 (*ClaHsp60*), 12 genes for Hsp70 (*ClaHsp70*), 6 genes for Hsp90 (*ClaHsp90*), and 102 genes for Hsp100 (*ClaHsp100*) were determined and numbered according to their chromosomal localization. It was determined that the length of the sHsps changed between 97 and 509 aa; most of them were acidic. The length of Hsp40 proteins changed between 60 and 2548 aa; 62 of the proteins were basic and 39 were acidic. It was seen that the protein lengths of the Hsp60s ranged from 57 to 1741 aa; 17 were acidic, 6 were basic. Amino acid content of Hsp70 proteins varied from 571 to 898 aa; all of them were acidic. It was found that the protein length of the Hsp90s was 699 to 811 aa long; all of them were acidic. Hsp100 proteins were 149 to 1911 aa in length, and 66 were acidic. The acidic property was dominant among Hsps when the results were considered. This can be attributed to the roles of Hsps in the cell. Detailed information including chromosomal localization, molecular weight, and instability index of watermelon Hsps are given in Supplementary material 2.

### 3.2. Chromosomal distribution and genetic structure of ClaHsp genes 

**Figure 1:**
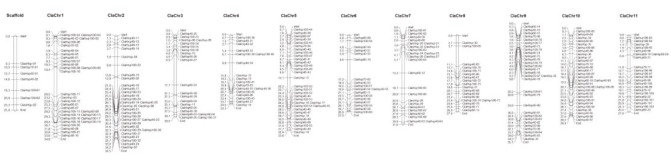
The localization of ClaHsp genes on watermelon chromosomes.

The genes identified for the Hsp family were distributed over 11 chromosomes in the watermelon (Figure 1). The majority of *ClasHsp* genes (10) were located on chromosome 7. There were 5 genes on the fifth and tenth chromosomes. When the chromosomal distribution of the *ClaHsp40* genes was examined, it was determined that the most genes (14 genes) were located on chromosomes 2 and 10, followed by chromosomes 5 and 9 with 12 genes (Figure 1). When the location of the *ClaHsp60* genes on the watermelon chromosomes was examined, 5 of the *Hsp60* genes were located on chromosome 11. The third, sixth, and ninth chromosomes had 3 *ClaHsp60* genes, the fifth and tenth chromosomes had 2 *ClaHsp60* genes, and other chromosomes had 1 each of the *ClaHsp60* genes (Figure 1). The *ClaHsp70* genes were located on chromosomes 1, 4, 5, 6, 9, 10, and 11. It appeared that the *ClaHsp90* genes were located on chromosomes 1, 2, 3, and 8. The majority of the *ClaHsp100* genes (19 of the *ClaHsp100* genes) were located on chromosome 1.

When the exon–intron structures of the analyzed *ClaHsp* genes were examined, it appeared that the exon and intron regions were approximately equal in *sHsp*, *Hsp40*, and *Hsp70* genes. *Hsp60* and *Hsp90 *genes were found to have entirely exon regions and no intronic regions. *Hsp100* genes had very few introns, mostly exons. Similarly, *Arabidopsis*, castor bean, rice, and sorghum were observed to have the same characteristics (Jakoby M et al., 2002; Nijhawan A et al., 2008; Jin Z et al., 2014; Wang J et al., 2011), suggesting evolutionary conservation. This can be attributed to their many functions in the cell. The exon–intron structures of the *ClaHsps* are shown in Supplementary material 3.

### 3.3. Phylogenetic tree analysis of ClaHsps and identification of protected motifs

**Figure 2:**
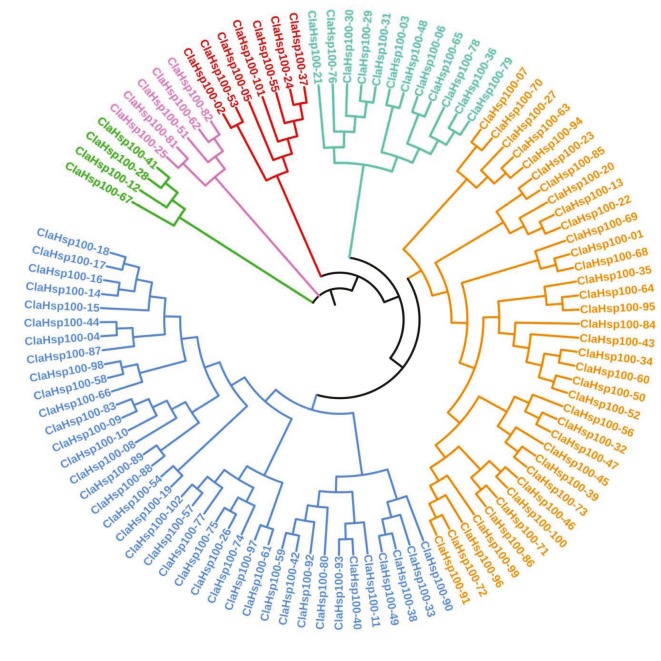
Phylogenetic tree of ClaHsp100 proteins. The phylogenetic trees of the Hsp families were drawn by multiple sequence alignment using the ClustalW program via the MEGA 7 program. The maximum likelihood method (Milligan, 2003) was used when phylogenetic trees were drawn. The drawn tree was visualized in iTOL database.

A phylogenetic tree was drawn and motif analysis was performed to evaluate the evolutionary relationships between the ClaHsps. When the phylogenetic trees drawn by maximum likelihood method were examined, it was seen that sHsp proteins divided into 4 main clusters (Clusters I–IV). It was determined that each cluster contained 7, 15, 6, and 11 proteins, respectively. The phylogenetic tree of ClaHsp40 proteins included 5 main clusters; 3, 3, 2, 5, and 88 proteins were detected in each cluster, respectively. The phylogenetic tree of ClaHsp60 proteins had 6 main clusters. According to phylogenetic analysis of ClaHsp70 proteins, 6 main clusters were formed. Four main clusters were defined for ClaHsp90 proteins. The ClaHsp100 proteins were divided into 6 main clusters in the phylogenetic tree. As a result, 4, 5, 7, 12, 36, and 38 proteins were found in each cluster, respectively (Figure 2). Each Hsp family was divided into a different number of subgroups (Supplementary material 4). The protected motifs obtained from the MEME suite database were analyzed. A total of 20 different motifs were identified for each Hsp family. When the motifs of ClasHsps were examined, 3 dominant motif patterns were observed. Seven motif patterns were determined among ClaHsp40 proteins. When the motif structure of ClaHsp60 was analyzed, the same motif structures were included in the proteins. Four motif patterns were shared among ClaHsp70 proteins, whereas ClaHsp90 proteins had 14 motif patterns. ClaHsp100 proteins had 6 dominant motif patterns. As a result, it has been determined that these genes contain motif patterns in accordance with the constructed phylogenetic trees (Supplementary material 5).

### 3.4. Ontology analysis of ClaHsp genes

**Figure 3:**
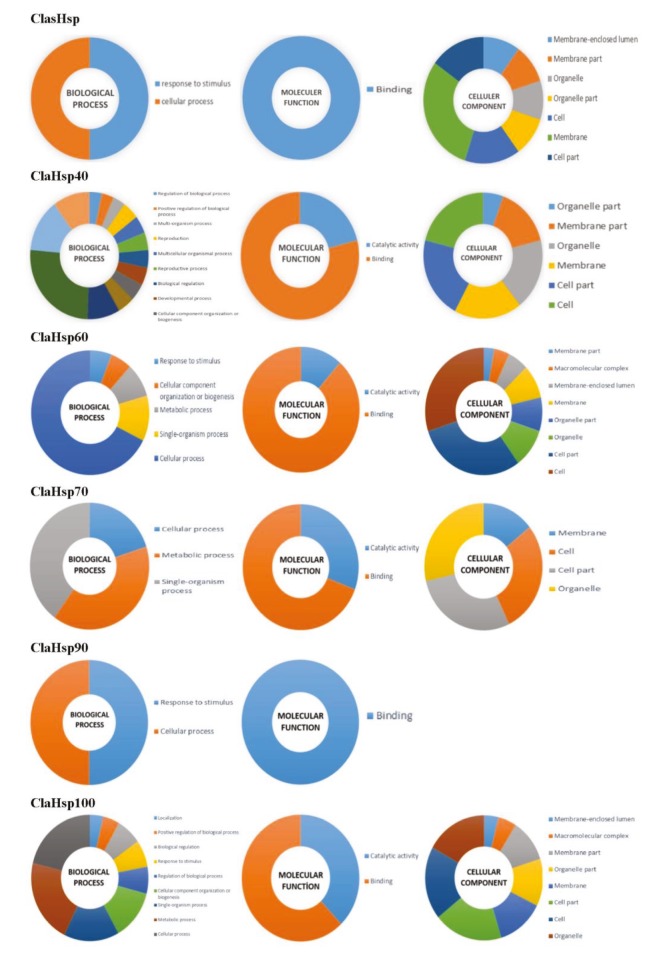
Gene ontology analysis of ClaHsp proteins.

Biological function, cellular localization, and molecular function of watermelon *Hsp* genes were determined by gene ontology analysis (Figure 3). sHsps have roles in cellular functions and response to stimuli, and are mainly found in the membrane, cell, and cell parts. It has been determined that Hsp40 proteins have variable biological functions such as regulation and positive regulation of biological processes, multiorganism processes, reproduction, multicellular organismal processes, biological regulation, cellular component organization, and biogenesis; the molecular function is binding and catalytic activity. ClaHsp40 family members were especially found in the organelles, membranes, cell, and cell parts. The Hsp60 ontology analysis showed that their biological function was mostly in cellular processes. Hsp60 was found to be the most abundant in the cell and cell parts. Hsp70 proteins had roles in cellular functions, metabolic processes, and single organism processes. They were located especially in the cell, cell parts, and organelles. Hsp90 proteins had 2 functions as a response to stimuli and cellular processes. Many biological processes including regulation of biological processes, response to stimuli, cellular component organization and biogenesis, cellular functions, and metabolic functions were determined for Hsp100 proteins. It was found that this protein group was mostly located in the membrane, cell parts, and organelles within the cell. It was observed that the molecular function of all ClaHsp proteins was achieved by binding activity.

### 3.5. Determination of orthologue genes and separation times of Hsp genes from watermelon and other plants

**Figure 4:**
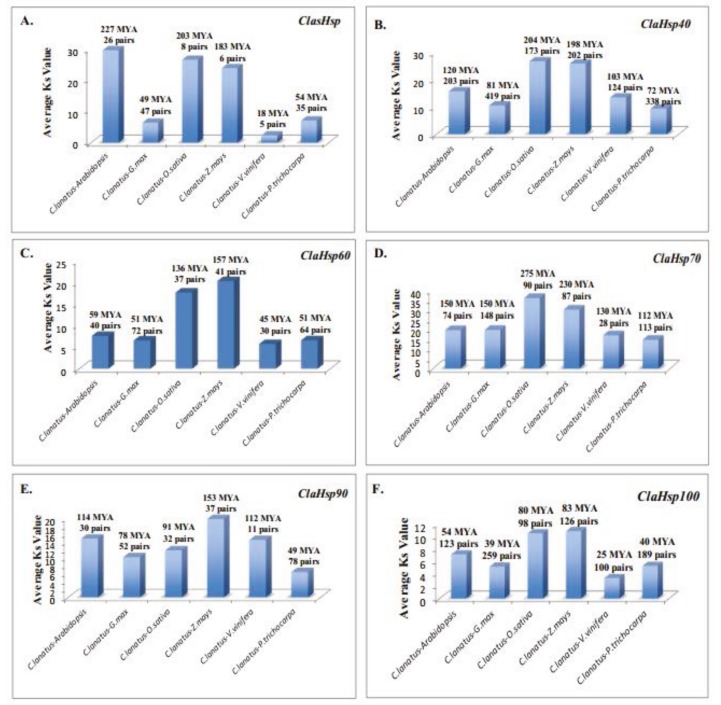
Orthologues of ClaHsp genes in other plants and divergence rates.

Sequences of *Hsp* gene families which were obtained from the watermelon genome were compared with sequences of different organisms (*Arabidopsis thaliana*,* Oryza sativa*,* Glycine max*,* Populus trichocarpa*,* Vitis vinifera*,**and *Zea mays*), and orthologous analysis was performed. As a result, orthologous gene pairs, similar and dissimilar rates of change, and probable differentiation times in the evolutionary process have been determined (Figure 4). According to the obtained data, the most orthologous relationship for the *ClasHsp* gene family was found to be with soybean (47 pairs) followed by poplar (35 pairs). The least orthologous relationships were observed with grape (5 pairs) and maize (6 pairs). The organisms with the highest similarity to watermelon among the 6 organisms for this gene family were soybean (0.05), grape (0.05), and poplar (0.05). The organism with the lowest similarity was identified as maize (0.02). When the divergence times were considered, it was determined that the first divergence with watermelon was by *Arabidopsis*, with an average of 227 million years ago (MYA). It was determined that the organism that last diverged from watermelon was grape, with 18 MYA for *sHsp* genes (Figure 4A). 

When the orthologous relationships between the *ClaHsp40* gene family and *Hsp40* genes from other plants were examined, it was observed that the most orthologous relationships were with soybean (419 pairs) and poplar (338 pairs). The least orthologous relationships with watermelon *Hsp40* genes were found with rice (173 pairs) and grape (124 pairs). The highest similarity ratio was found with soybean (0.11) and poplar (0.10) *Hsp40* genes. The least similarity was determined between rice and watermelon with 0.03 similarities. When the possible separation times were examined, it was observed that the first separation was with rice about 204 MYA. It was then separated from maize about 198 MYA. It was observed that the latest separation was with poplar, about 72 MYA (Figure 4B).

When the orthologous relationships of the *ClaHsp60* gene family were examined, it was observed that the most orthologous relationship was with soybean, with 72 pairs. This was followed by poplar with 64 pairs. The orthologous relationships with maize and *Arabidopsis* included 41 and 40 pairs, respectively. In the *ClaHsp60* genes, the organisms which had the least orthologous relationship with watermelon were rice and grape with 37 and 30 pairs, respectively. Grape had the highest similarity ratio with watermelon with 0.05. The similarity ratio between watermelon and soybean was 0.04. This was followed by *Arabidopsis* and poplar with 0.03 and 0.25, respectively. The organisms which had the lowest similarity ratio with watermelon were rice and maize with 0.02. When the estimated divergence times with these organisms were considered, the first separation was with maize 157 MYA. It was observed that the last separation was with soybean and poplar about 51 MYA (Figure 4C).

When the orthologous relationships of the *ClaHsp70* gene family were evaluated, it was determined that the most orthologous relationship was between watermelon and soybean (148 pairs). This relationship was followed by poplar with 113 pairs. The least orthologous relationship was between watermelon and grape with 28 pairs. When the similarity rates with these organisms were examined, *Arabidopsis*, rice, and maize were found to have almost the same similarity ratio (0.01). The grape had the highest similarity ratio (0.03). Considering divergence times, the earliest diverged genes from watermelon were rice genes, with an average of 275 MYA. The latest divergence was determined to be with poplar, 112 MYA (Figure 4D).

When the orthologous relationships of *ClaHsp90* genes were analyzed, the most orthologous relationship was with poplar with 78 pairs. This was followed by soybean, with a highly orthologous relationship with 52 pairs. The least orthologous relationship was found with grape (11 pairs). When similarity ratios were examined, it was determined that watermelon and *Arabidopsis*, soybean, grape, and poplar had almost the same similarity rate. Rice and maize had about the same similarity ratio with watermelon. Considering divergence times of *ClaHsp90* genes, the first diverged genes from *ClaHsp90* genes were maize genes, with an average of 153 MYA. It was estimated that the most recent divergence was with poplar 49 MYA (Figure 4E).

Finally, when considering the orthologous relationships of *ClaHsp100* genes with other organisms, it was found that the most orthologous relationships were**between watermelon and soybean (259 pairs) and watermelon and poplar (189 pairs). These were followed by maize (126 pairs) and *Arabidopsis* (123 pairs). The least orthologous relationships were with grape and rice with 100 and 98 gene pairs, respectively. When the similarity ratios of these organisms with watermelon were calculated, it was determined that grape and poplar had the highest similarity ratio (0.06). It was then observed that similarity rates for soybean and *Arabidopsis* were 0.05 and 0.04, respectively. The lowest similarity ratio was found to be with rice and maize (0.03). When the estimated divergence times were examined, the first separation was with maize (83 MYA). The latest organism to separate from watermelon was grape (25 MYA) (Figure 4F) (Supplementary material 6).

### 3.6. Homology modeling of ClaHsps

Information about the predicated 3-dimensional structures of ClaHsps were obtained by homology modeling using the Protein Data Bank (PDB). When sHsps were examined, 20 ClasHsp proteins showed high similarity. The similarity rate was determined by selecting the intensive mode from the Phyre2 database. Reliability was 90%, and similarity was between 70% and 100%. As a result, it was seen that β-sheet structure predominated in the 3D structures of ClasHsps. When the other Hsp family members were analyzed, 4 proteins for the ClaHsp40 family, 15 proteins for the ClaHsp60 family, 9 proteins for the Hsp70 family, 3 proteins for the Hsp90 family, and 38 proteins for the Hsp100 family showed high similarity. Alpha helix structure was dominant among all of these analyzed proteins, and a few β-sheet structures were determined, except for ClaHsp40 proteins. Detailed figures are given in the Supplementary material 7.

### 3.7. Determination of miRNAs targeting ClaHsp genes

**Figure 5:**
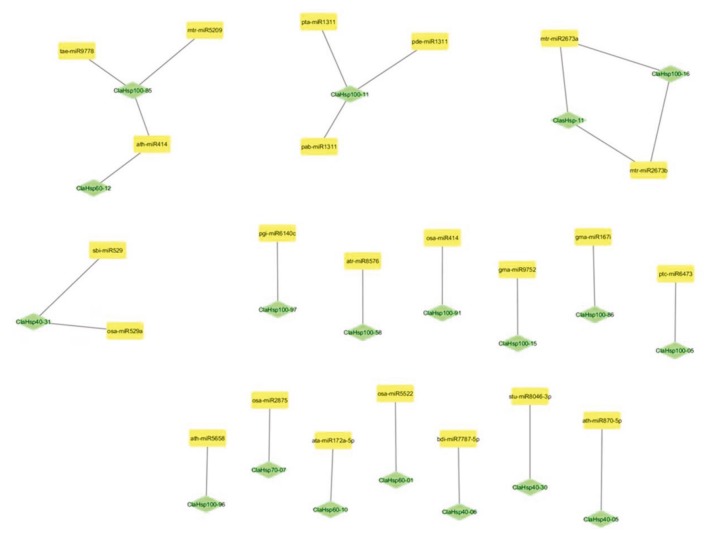
ClaHsp genes targeted by miRNAs.

As a result of miRNA analysis using the psRNA Target Server (http://plantgrn.noble.org/psRNATarget/), it was determined that a total of 19 different watermelon *Hsp* genes were targeted by miRNAs. *ClasHsp-11*, *ClaHsp40-05*, *ClaHsp40-06*, *ClaHsp40-30*, *ClaHsp40-31*, *ClaHsp60-01*, *ClaHsp60-10*, *ClaHsp60-12*, *ClaHsp70-07*, *ClaHsp100-05*, *ClaHsp100-11*, *ClaHsp100-15*, *ClaHsp100-16*, *ClaHsp100-58*, *ClaHsp100-85*, *ClaHsp100-86*, *ClaHsp100-91*, *ClaHsp100-96*, and *ClaHsp100-97* genes were targeted by a total of 23 different miRNAs. *ClaHsp* genes targeted by miRNAs are shown in detail in Figure 5.

### 3.8. In silico gene expression analyses and expression profiles of ClaHsp genes under combined drought and temperature stresses 

To determine the expression of the watermelon *Hsp* genes, individual heat maps for each Hsp family were drawn using SRR files. As a result of SRA analysis, expression levels under various stress conditions in different tissues of the watermelon were examined. Heat maps containing different tissues and fruits are shown in Supplementary material 8. According to the results, *ClasHsp-23* gene was upregulated in the**floem and vascular tissues and on the 10th, 18th, 26th, 28th, and 34th days of fruit development after pollination. There was an increasing pattern in the expressions of *ClaHsp40-15* and *ClaHsp40-96 *genes on the 18th, 26th, 28th, 34th, 42nd, and 50th days of fruit development stages, as well as in vascular and floem tissues. Additionally, *ClaHsp60-09* and *ClaHsp60-15 *genes have an increase profile in all studied stages of fruit development. In addition, *ClaHsp70-07*, *ClaHsp70-10*,* ClaHsp90-04*,**and *ClaHsp90-05 *genes were upregulated on the 18th, 26th, 28th, 34th, 42nd, and 50th days of fruit development and in vascular and floem tissues. Moreover, the expressions of *ClaHsp100-54 *and *ClaHsp100-67* genes were induced on the 18th, 28th, 34th, 42nd, and 50th days of fruit development stages and in vascular and floem tissues. 

Based on the transcriptome results, combined drought and temperature stress responses were tested for in selected *Hsp* genes from watermelon using qRT-PCR. For each Hsp family, qRT-PCR analyses were performed using root, stem, leaf, and tendril samples, and time-dependent expression graphs were drawn (Supplementary material 9). When the expression levels of the watermelon stem sample were examined, it was observed that there was an increase pattern at 30 min of combined stress application in the expression levels of all of the Hsp families (*ClasHsp-23*, *ClaHsp40-15*, *ClaHsp60-15*, *ClaHsp70-07*, *ClaHsp90-04*, *ClaHsp100-67*). Subsequently, it was determined that the expressions levels decreased during the first hour and increased again during the second hour. 

When the watermelon leaf sample was examined, it was observed that there was a certain increase in the *ClasHsp-23* gene from the first hour. A significant increase was observed for the *ClaHsp40-15*, *ClaHsp60-15*, and *ClaHsp100-67* genes for the first 30 min, and a decrease in the following hours was determined. It was also observed that the maximum expression for the *ClaHsp60-15* and *ClaHsp100-67* genes was at 30 min. For the *ClaHsp70-07* and *ClaHsp90-04* genes, it was determined that the first reaction to stress was in the first hour. When watermelon tendril samples were viewed, it was detected that the expressions of the *ClasHsp-23*, *ClaHsp60-15*, *ClaHsp70-07*, *ClaHsp90-04*, and *ClaHsp100-67* genes were upregulated at 30 min, then downregulated at 1 h, and increased again at 2 h. In the tendril samples, the highest response against combined drought and temperature stress was observed at 30 min for the *ClaHsp70-07* and *ClaHsp90-04* genes.

When the watermelon root samples were examined, it was observed that there was an increase pattern in the *ClaHsp40-15*, *ClaHsp90-04*, and *ClaHsp100-67* genes up to 30 min and then the expression was decreased. It was determined that in the *ClasHsp-23* and *ClaHsp70-07* genes, expression increased at 30 min, followed by a decrease in the first hour and a rise in the second hour. The highest expression level in the *ClaHsp60-15* gene was observed at the first hour. 

**Figure 6:**
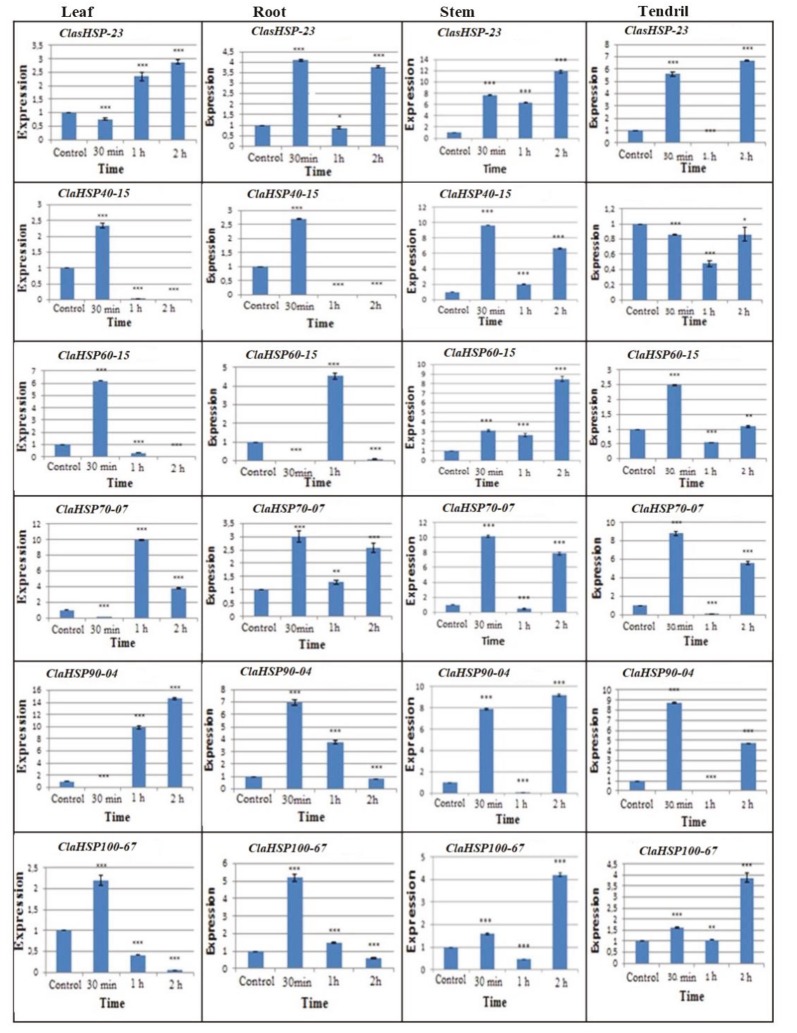
The expression levels of ClaHsp genes in stem, root, leaf, and tendril tissues of watermelon under combined drought and temperature stresses at 0 (control), 30 min, 1 h, and 2 h.

When all the results were evaluated, a rapid increase pattern was seen in the first 30 min of combined drought and temperature stresses. The expression levels of all of the genes studied in the watermelon stem tissues thereafter decreased at the first hour and increased again in the second hour. In the tendril tissue samples, this expression period was commonly observed in *ClasHsp-23*, *ClaHsp60-15*, *ClaHsp70-07*, *ClaHsp90-04*,**and *ClaHsp100-67* genes. In stressed root samples, this period was observed in the *ClasHsp-23* and *ClaHsp70-07* genes. The *ClasHsp-23* and *ClaHsp70-07* genes can be described as the first genes to respond to the stress in the stem, root, and tendril tissues. In addition, *ClaHsp100-67* gene expression showed a rapid increase in the first 30 min of the stress application in all studied tissues. For this reason, the *ClaHsp100-67* gene may be considered an early response gene which responds to combined drought and temperature stresses in all tissues (Figure 6).

## 4. Discussion

Researching the response of plants to a combination of different stresses can be helpful for elucidating stress response mechanisms in plants, because plants are often exposed to a combination of stresses in nature, including drought and heat shock. 

In this study, the changes in the expression profiles of watermelon *Hsp* genes against high temperature and drought stress combinations were examined, and the response to these stresses was interpreted. As a result of bioinformatics analysis, genes belonging to each Hsp family were determined in the watermelon genome. The Hsp40 and Hsp100 families had the highest number of genes with 101 and 102 genes, respectively. After that, 39 genes belonging to the sHsp family and 23 genes belonging to the Hsp60 family were found. The Hsp families containing the lowest number of genes in the watermelon genome were Hsp70 and Hsp90 with 12 and 6 genes, respectively.

It is an expected situation that the number of Hsp40 proteins acting as cochaperones of the Hsp70 protein is higher than that of Hsp70. A total of 283 *ClaHsp* genes were identified in the watermelon genome, located over 11 watermelon chromosomes. The most *ClaHsp* genes were on the fifth chromosome, and the lowest number of *Hsp* genes were on the eleventh chromosome. The genes belonging to the Hsp family in the watermelon genome have been identified for the first time in this study. In the literature, similar gene numbers have been observed in other organisms when compared to the *Hsp* genes detected in the watermelon genome. In a study conducted by our research group, it was determined that 60 sHsp, 145 Hsp40, 49 Hsp60, 34 Hsp70, 12 Hsp90, and 90 Hsp100 genes were identified in the poplar genome (Yer et al., 2016; Yer et al., 2018). Members of the Hsp70 family are the most thoroughly studied group of stress proteins up to the present. As a result, 32 genes in the rice (Sarkar, 2013), 21 genes in the eucalyptus (Çelik Altunoğlu, 2016a), 18 genes in the *Arabidopsis* (Lin et al., 2001; Sung et al., 2001), 13 genes in the beech, 17 genes in the oak, and 15 genes in the chestnut (Yer et al., 2016) genomes have been identified as *Hsp70* genes. Other studies on other Hsp family members have identified 29 *Hsp60* genes (Hill and Hemmingsen, 2001), 7 *Hsp90 *genes (Krishna and Gloor, 2001), and 13 *sHsp* genes (Scharf et al., 2001) when researching the *Arabidopsis thaliana* genome. Furthermore, 23 sHsps (Sarkar et al., 2009), 104 Hsp40 (Sarkar et al., 2013), 20 Hsp60 (Wang et al., 2014), 9 Hsp90, and 10 Hsp100 (Hu et al., 2009) proteins were determined in the rice genome. According to our results, the most numerous Hsp family members in the watermelon genome were Hsp40 and Hsp100. This was similar with poplar for Hsp40 and Hsp100, and rice for Hsp40. Moreover, Hsp100/ClpB proteins have an important role in tolerance to high temperatures in various organisms (Hong and Vierling, 2001). Therefore, it is an expected situation to have a large number of *Hsp100* genes in watermelon growing in a hot climate. 

Exon–intron structures have been determined in order to examine the structure of the *ClaHsp* genes. In the ClasHsp, ClaHsp40, and ClaHsp70 families, exons–introns were approximately equal, while very few introns were detected in ClaHsp100 and no introns were observed in the ClaHsp60 and ClaHsp90 families. When the other studies were examined, it was seen that the exon structure predominated in the *sHsp*, *Hsp70*, and *Hsp100* genes of the Hsp families in the *A. thaliana* genome (Zhang et al., 2015). When the Hsp70 family in the eucalyptus genome was examined, it was found that there were an approximately equal number of exon–intron structures as in the watermelon Hsp70 family (Çelik Altunoğlu, 2016a). Genes with the same physical structure, such as exon–intron length, were located in the same cluster in the phylogenetic tree. It can be concluded from this that the phylogenetic tree distribution in each cluster is consistent with the exon–intron structures. 

Molecular and biological functions and localization of every Hsp family in the cell were determined by analyzing the gene ontology of ClaHsps. As a result, it was seen that the molecular function of each Hsp family was predominantly binding. Their biological functions were often as a response to stimuli; they were also involved in cellular and metabolic processes and the regulation of biological functions. It was observed that ClaHsps were localized in different parts of the cell. They were mainly found in the inner parts of the cell, membranes, and organelles. When other studies in the literature were examined, it was found that Hsp families were mostly located in intracellular parts in eucalyptus (Çelik Altunoğlu, 2016) and poplar (Zhang et al., 2015; Yer et al., 2016; Yer et al., 2018). The results for watermelon Hsp proteins have parallels with the results of these studies. These localizations may be the reason for the multiple roles of these family members in organisms. 

By comparing the *Hsp* genes in the watermelon genome with those of other organisms, we have identified orthologous genes that are different in structure but functionally similar. We gathered information about the evolutionary processes of *ClaHsp *genes and the orthologous relationships between watermelon and *Arabidopsis*, soybean, rice, poplar, grape, and maize. In the majority of *ClaHsp* gene families apart from *ClaHsp90*, the most orthologous relationship appeared to be between watermelon and soybean; this was closely followed by poplar. For *ClaHsp90* genes, the most orthologous genes were defined with poplar and then with soybean. The least orthologous relationship was found between watermelon and grape. When the similarity rates were examined, high similarity was observed between watermelon and soybean and grape *Hsp* genes. It has been determined that maize had the least similarity with watermelon. The first organism that separated from watermelon was usually maize or rice for the *ClaHsp* gene families. When the results of these analyses were considered, it was thought that *Hsp* genes in the watermelon and in the poplar diverged later than the *Hsp* genes in other organisms. Therefore, the differentiation was lower with the poplar. Another study which reported the high similarity between the watermelon and the poplar *LEA* genes was also performed by our group. It has also been observed that the most recent divergence time for *LEA* genes was between watermelon and poplar (Çelik Altunoğlu, 2017). In addition, the earliest divergence of *Hsp* genes in the poplar genome was determined with the rice genome, as for many watermelon *Hsp* genes (Yer et al., 2018). These results may help achieve better understanding of the evolutionary relationship between these organisms and the watermelon based on the *Hsp* genes. Therefore, it is thought that more efficient results can be obtained in future plant breeding and cloning studies by using this evolutionary relationship. 

When the predicated 3D structure of proteins was examined, it was seen that β-sheet structure predominated in ClasHsps. However, the alpha helix structure was dominant among all other analyzed proteins and a few β-sheet structure were determined, except in ClaHsp40 proteins.****This structural feature could allow the proteins to bind firmly to the substrate binding domain (SBD). It could be said that α-helix and β structures are dominant in molecular functions and that therefore they are an important form of structure. The determination of these structural properties provides an idea for determining the structure, function, and mutation relationships of proteins (Mayer et al., 2001). In addition, this structure may contribute to fluid loss and alleviate potential damage in plant wounding or stress (Li, 2015).

When miRNAs targeting these *Hsp* genes in the watermelon genome were examined, the miRNAs targeting the *ClasHsp-11* gene were mtr-miR2673a and mtr-miR2673b. In the literature, miR2673 appeared as a miRNA associated with drought (Yang et al., 2013). In the ClaHsp40 family, *ClaHsp40-05*, *ClaHsp40-06*, *ClaHsp40-30*, and *ClaHsp40-31* genes were targeted by ath-miR870-5p, bdi-miR7787-5p, stu-miR8046-3p, sbi-miR529, and osa-miR529a miRNAs. The *ClaHsp60-01*, *ClaHsp60-10*, and *ClaHsp60-12* genes of the ClaHsp60 family were targeted by osa-miR5522, ata-miR172a-5p, and ath-miR414 miRNAs. It has been determined that miR172 is associated with drought stress and the coding of transcription factors for flower development in *Arabidopsis*. Therefore, it has been concluded that miRNA plays significant roles in plant growth, metabolism, and response to stress (Yang et al., 2013). In addition, other studies for miR414 have found that miR414 has an important role in the regulation of plant growth and development (Guleria et al., 2011). It was determined that the miRNA targeting the *ClaHsp70-07* gene from the ClaHsp70 family was OsA-miR2875. We have not found an miRNA that targets genes belonging to the ClaHsp90 family. *ClaHsp100-05*, *ClaHsp100-11*, *ClaHsp100-15*, *ClaHsp100-16*, *ClaHsp100-58*, *ClaHsp100-85*, *ClaHsp100-86*, *ClaHsp100-91*, *ClaHsp100-96*, and *ClaHsp100-97* genes were targeted by miR6473, miR1311, miR414, miR2673, miR8576, miR5209, miR9778, miR167i, miR5658, and miR614c miRNAs. The ClaHsp100 family had the *Hsp* genes most often targeted by miRNAs. In total, 19 different watermelon *Hsp* genes were targeted by 23 different miRNAs. Determination of miRNAs targeting *ClaHsp* genes can help to resolve their functions in the watermelon.

In addition, combined drought and temperature stresses were applied and expression levels of *Hsp* genes were examined by qRT-PCR analysis. Overall, the results of these analyses conspicuously showed that the majority of the Hsp families displayed high expression levels at 30 min in all of the studied watermelon tissues (root, stem, leaf, and tendril). This is thought to be the first reaction against stress. The level of expression then generally decreased at the first hour and increased again during the second hour of stress. It is thought that the Hsp gene increase at 30 min was not enough, and thus it was augmented again in the second hour. In a study on *Arabidopsis*, it was observed that the expression levels of some *Hsp70* genes were upregulated 2–20 times as from 30 min under 40 °C heat stress (Sung et al., 2001). In another study based on profiles of Arabidopsis heat shock proteins (Hsp) and transcription factors (Hsf), Hsf and Hsp expression was strongly induced by heat, cold, salt, and osmotic stress, while other types of stress including drought, genotoxic stress, ultraviolet light, oxidative stress, wounding, and pathogen infection induced family or tissue-specific response patterns (Swindell et al., 2007). In rice, it was detected that some sHsp transcripts started to increase in a short time as 5 min under 41 °C heat stress (Guan et al., 2004). When considering the expressions of genes orthologous**to *ClaHsp* genes in poplar, the *PtHsp70-09* gene with the number of Potri.003G006300.1, which was orthologous to *ClaHsp70-07*, and *PtHsp90-12* gene with the number of Potri.017G146600.1, which was orthologous to *ClaHsp90-04*, displayed an increase pattern after salt stress application in various poplar clones (Yer et al., 2018). In addition, considering RNA sequence data by Tang et al., *PtHsp70-10* with the number of Potri.004G224400.1 and *PtHsp70*-*20* with the number of Potri.009G079700.1, which were orthologous of *ClaHsp70-07*, were induced under drought stress conditions in the leaf tissues of poplar (*Populus trichocarpa)* (Tang et al., 2015).**It can be concluded that the *ClaHsp70-07 *gene orthologous in poplar has a response against drought and salt stress conditions. *ClaHsp90-04 *had orthologous genes in *Arabidopsis* with the numbers of AT5G52640, AT5G56000, and AT5G56010 which have been reported to be heat- and drought-responsive genes in this plant. In addition,**AT5G09590, which was orthologous to the *ClaHsp70-07* gene, was related with drought and heat reaction in *Arabidopsis* (Ghatak et al., 2016). According to our study, *ClaHsp70-07* genes may be the first response genes to the stress in the stem, root, and tendril tissues of watermelon under combined drought and heat stresses.**In addition, the *ClaHsp100-67* gene displayed a rapid increase in the first 30 min of stress application in all of the studied tissues of watermelon, and thus may be considered an early response gene under combined drought and temperature stresses. It is understood that the increase in expression levels of the Hsp families in our study is consistent with those studies based on stress responses. It has been determined that there is a relationship between these *Hsp* genes in watermelon and drought–temperature stress according to the gene expression results. This relationship was also supported by bioinformatics analyses. Considering the changes in expression levels, it is thought that the functions of the mentioned genes can be understood better. 

## 5. Conclusion

The current study provides a detailed genomewide determination of Hsp family members in the watermelon genome, which can help resolve the functions of Hsps. Considering the results and the increase of climate change in the world, this study may be the first step towards the development of high-tolerance watermelon plants, whose products are among the 5 most consumed fresh fruits around the world, against future drought and temperature stresses. In addition, the study contains important literature information for other studies to be done in the future.

**Acknowledgments**

This work was completely supported by the Kastamonu University Scientific Research Project Coordinatorship with the project code of KUBAP-01/2016-45.
